# Multicystic peritoneal mesothelioma: a systematic review of the literature

**DOI:** 10.1515/pp-2019-0024

**Published:** 2019-09-25

**Authors:** Barbara Noiret, Florence Renaud, Guillaume Piessen, Clarisse Eveno

**Affiliations:** Claude Huriez University Hospital, Lille, France; Centre de Recherche Jean-Pierre Aubert, Lille, Hauts-de-France, France; UMR 1172, Lille, Hauts-de-France, France; Institut de pathologie, CHRU de Lille Pôle Biologie-Pathologie-Génétique, Lille, Hauts-de-France, France

**Keywords:** cytoreductive surgery, diagnosis, hyperthermic intraperitoneal chemotherapy, multicystic peritoneal mesothelioma, pathogenesis

## Abstract

Multicystic peritoneal mesothelioma (MCPM) is a particularly rare and benign neoplasm that arises from the peritoneum in reproductive aged females. Its etiopathogenesis is still unclear. The current prevailing theory supports the idea that the tumor is the result of an excessive inflammatory process. Because of a lack of clinical and imaging presentation, the diagnosis is intricate, and heavily relies on case reports and short studies. A histological analysis with immunohistochemistry is required for a definitive diagnosis. To date, there is no standard treatment recommended for MCPM. However, some studies suggest proceeding with a cytoreductive surgery and a hyperthermic intraperitoneal chemotherapy combining CISPLATIN and DOXORUBICIN, due to a high incidence of recurrence rate after medical treatment or surgery alone and potential malignant transformation.

## Introduction

Peritoneal mesothelioma is a particularly rare disease characterized by peritoneal malignancy occurring in the mesothelium (lining cells) of the pleural, peritoneal and pericardial cavities, and tunica vaginalis covering the testes. Seven histological types have been identified thus far: low-grade mesothelioma, well differentiated papillary, multicystic, epithelioid, sarcomatoid, biphasic (combining epithelioid and sarcomatoid), and deciduoïd [1]. Multicystic peritoneal mesothelioma (MCPM) accounts for 3 to 5% of peritoneal mesotheliomas and the estimated incidence is 2 for 1,000,000 per year [2]. This tumor was originally described by Plaut in 1928 as a cyst of the pelvis after being discovered by accident during a surgery of uterine leiomyomas. In 1979, Mennemeyer and Smith first defined the lesion as a « multicystic peritoneal mesothelioma » in a 27-year-old female with multicystic diffuse lesion involving omentum, peritoneum, and pelvic viscera [3]. Over 200 cases have been reported worldwide in 2017. MCPM occurs mainly in young to middle-aged women [4] at an average age of 37 years [5]. MPCM is classically found in the pelvis. The lesion is usually described as a benign tumor with a low risk of malignant transformation. However, its origin and pathogenesis remain controversial due to the rarity of the disease. There is to date no pathognomonic clinical and imaging requirement to clarify the diagnosis, and the definitive diagnosis is solely based on histology. Several therapeutic approaches for MCPM have been reported from simple observations to complete surgical resections, but the commonly accepted strategy combines cytoreductive surgery (CRS) and hyperthermic intraperitoneal chemotherapy (HIPEC).

## Pathogenesis

As the pathogenesis is still unclear, current understanding of the disease is relies heavily on case reports and short series. In contrast to malignant peritoneal mesothelioma, the prevalence of the tumor has no association with asbestos exposure. The prevailing theory suggests that MCPM may be a peritoneal reaction secondary to a chronic irritant with mesothelial cell entrapment, reactive proliferation, and cystic formation [6]. Chronic inflammation, previous surgery procedures, endometriosis, or recurrent peritonitis episodes associated with peritoneal dialysis [7], are described in the literature as predisposing factors of MCPM [8, 9]. Small foci of endometriosis have also been found in MCPM cystic walls and adjacent to endometriosis cysts in the pelvic space [10]. These histological findings suggest that endometriosis may contribute to the origin of MCPM. Other authors, on the contrary, put forward the hypothesis of a neoplastic process as the initiator of MCPM in which benign tumor grows slowly and can turn to malignant tumor. The authors suggest classifying the benign MCPM as low-grade or borderline disease [11].

In addition, some studies suggest that female sex hormones play a role in its pathogenesis. This hypothesis is supported by the fact that MCPM occurs mainly in females of reproductive age. High CA 19.9 serum concentration has been associated with the diagnosis of MCPM [12]. Finally, the tumor is rarely associated with an adenomatoid tumor (which is a benign neoplasm of mesothelial cells, most commonly occurring in the fallopian tube, uterus, and epididymis) with a close histogenetic relationship. Some tumors have been described with two associated components: adenomatoid and multicystic [13], which suggests that MCPM might present the characteristics of a borderline lesion between an adenomatoid tumor and a malignant mesothelioma.

## Diagnosis

The diagnosis is usually difficult to establish based on clinical and imaging data. The majority of patients are typically asymptomatic until the tumor is large enough to cause a mass effect on organs. The most common presenting symptoms are non-specific such as abdominal pain, tenderness, palpable mass, or weigh loss. The lesion shows a strong predilection for the pelvic peritoneum, which may adhere to the rectum, bladder, or uterus. Consequently, the other symptoms may be: dyspareunia, dysuria, intestinal obstruction, urinary symptoms, although these symptoms remain non-specific [14].

The preoperative diagnosis is challenging. Ultrasonography (US) is often the first examination requested in case of abdominal pain, due to its accessibility and the absence of irradiation source. Multiloculated anechoic cysts with liquid content can be observed on US. A “spider-in-web” sign can be described as an ovary encased by cystic mesothelioma of peritoneum [14].

CT scan is used to evaluate the location and the extent of the cystic mass, where lesion appears as a low-density, multi-loculated, and thin-walled multicystic mass [15]. However, the results obtained by US and CT-scans do not differentiate MCPM from other cystic mass.

**Figure 1:**
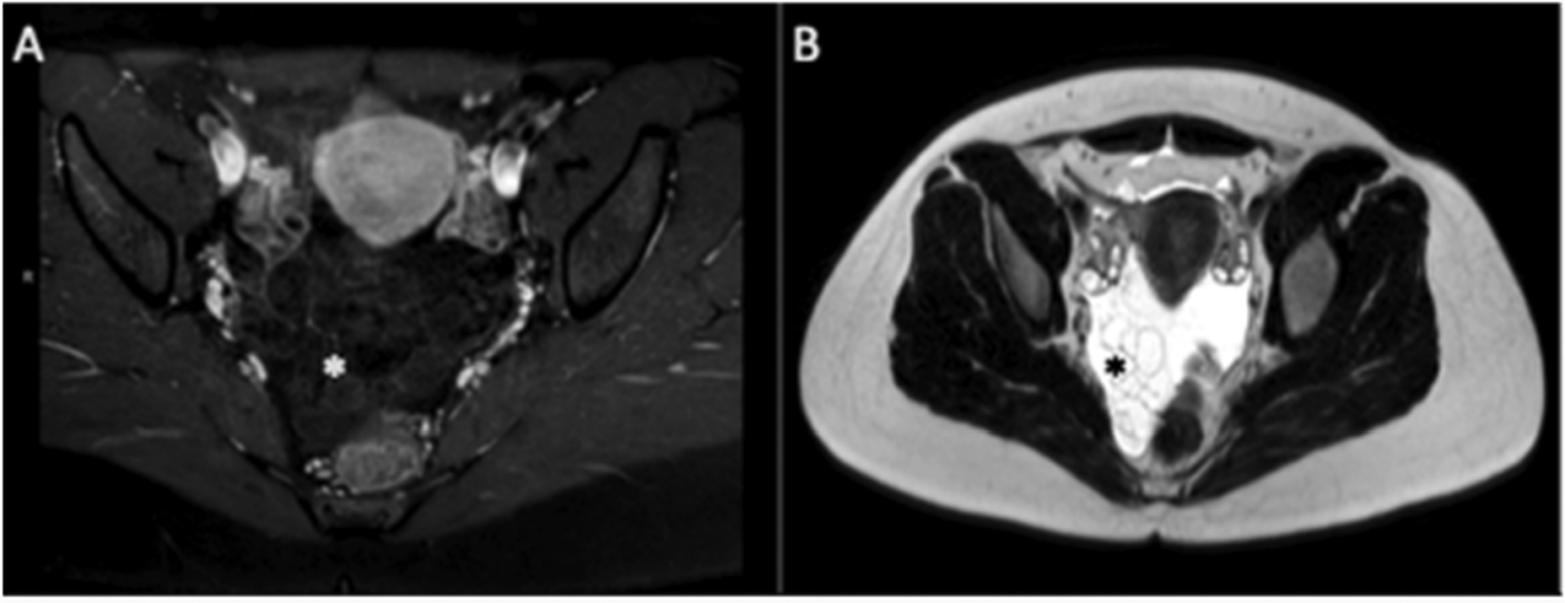
Multicystic mass (star) located in the pelvis with a decreased signal on T1-weighted image (A) but an increased signal intensity on T2-weighted image (B).

MRI is considered the best imaging technique. MCPM appear as hypointense lesions on T1 and hyper to intermediate intense on T2, with a mild contract enhancement of the wall [16] [Figure 1A, B]. However, hemorrhagic cysts may appear as hyperintense lesions on T1 and hypointense on T2. The mean diameter of MPCM is 13 cm at the time of diagnosis [17].

Lymphangioma has the most important differential imaging diagnosis, but it occurs mainly in the pediatric population and do not have a gender or regional predilection [18].

However, a definitive diagnosis requires histology as well, and the intraoperative examination is unnecessary since the diagnosis requires immunohistochemistry.

**Figure 2:**
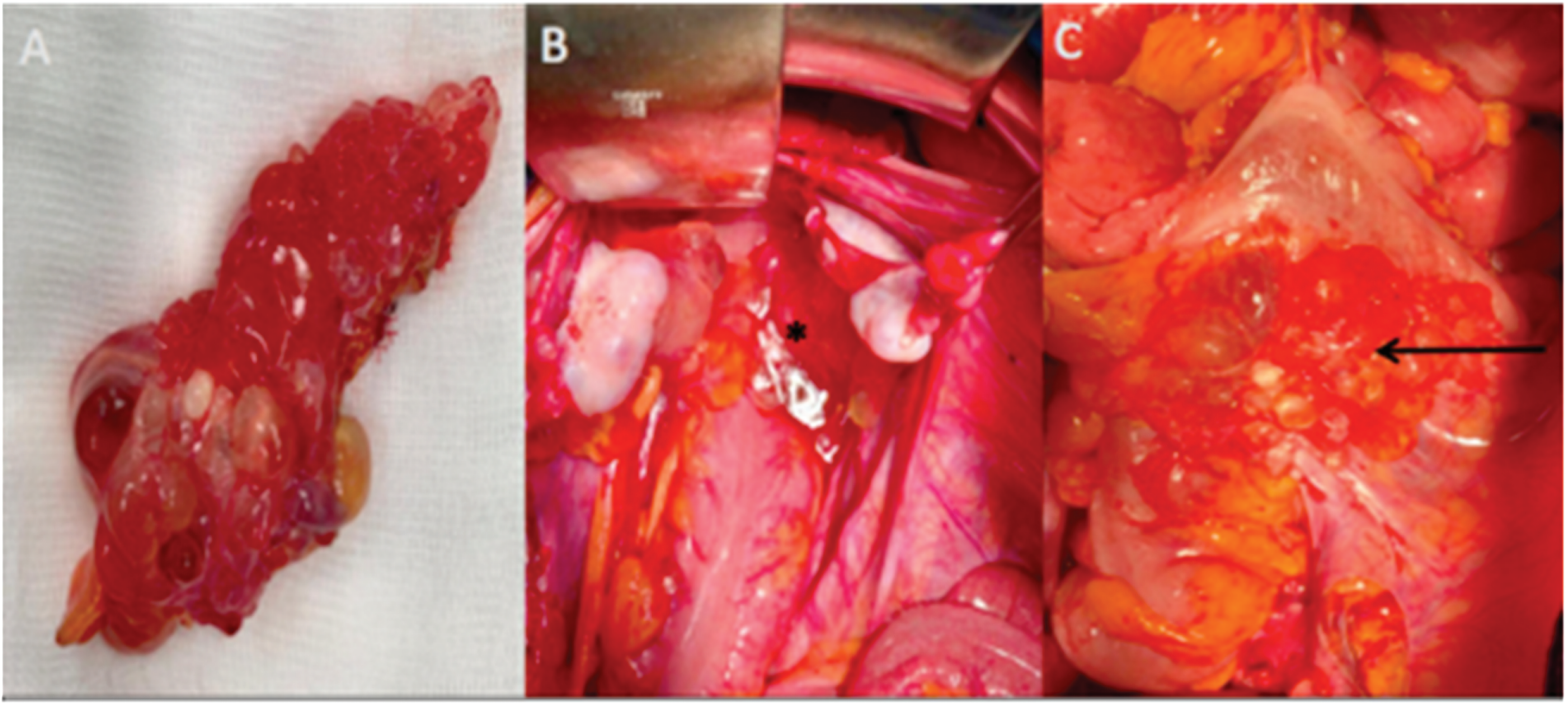
Multicystic mass is composed of multiple translucent cysts gathered in a grape-like form (A), filled with serous fluid, hemorrhagic or gelatinous materials. Multicystic in douglas pouch (star), adherent to the rectum (arrow).

Laparoscopy is the most efficient procedure to obtain the definitive diagnosis of MCPM and to analyze peritoneal extension of the disease, using large surgical biopsies. Macroscopically, the tumor is composed of multiple mesothelial and thin-walled translucent cysts ranging from several millimeters to 20 centimeters, arranged in a grape-like form. Cysts are mostly composed of clear serous fluid and occasionally mucinous, gelatinous, or hemorrhagic fluid [Figure 2A, B, C]. Microscopically, the cysts show typical morphologic features: multiple mesothelium-lined cystic spaces surrounded by a delicate thin fibrovascular stroma. The cystic lesions are lined by a single layer of flattened or cuboidal regular mesothelial cells [17].

The differential diagnosis of MCPM includes benign and malignant cystic or multicystic abdominal tumors such as cystic lymphangioma, endometriosis [19], cystic forms of endosalpingiosis [20], cystic adenomatoïd tumor, pseudomyxoma peritonei (PMP), malignant peritoneal mesothelioma.

**Figure 3:**
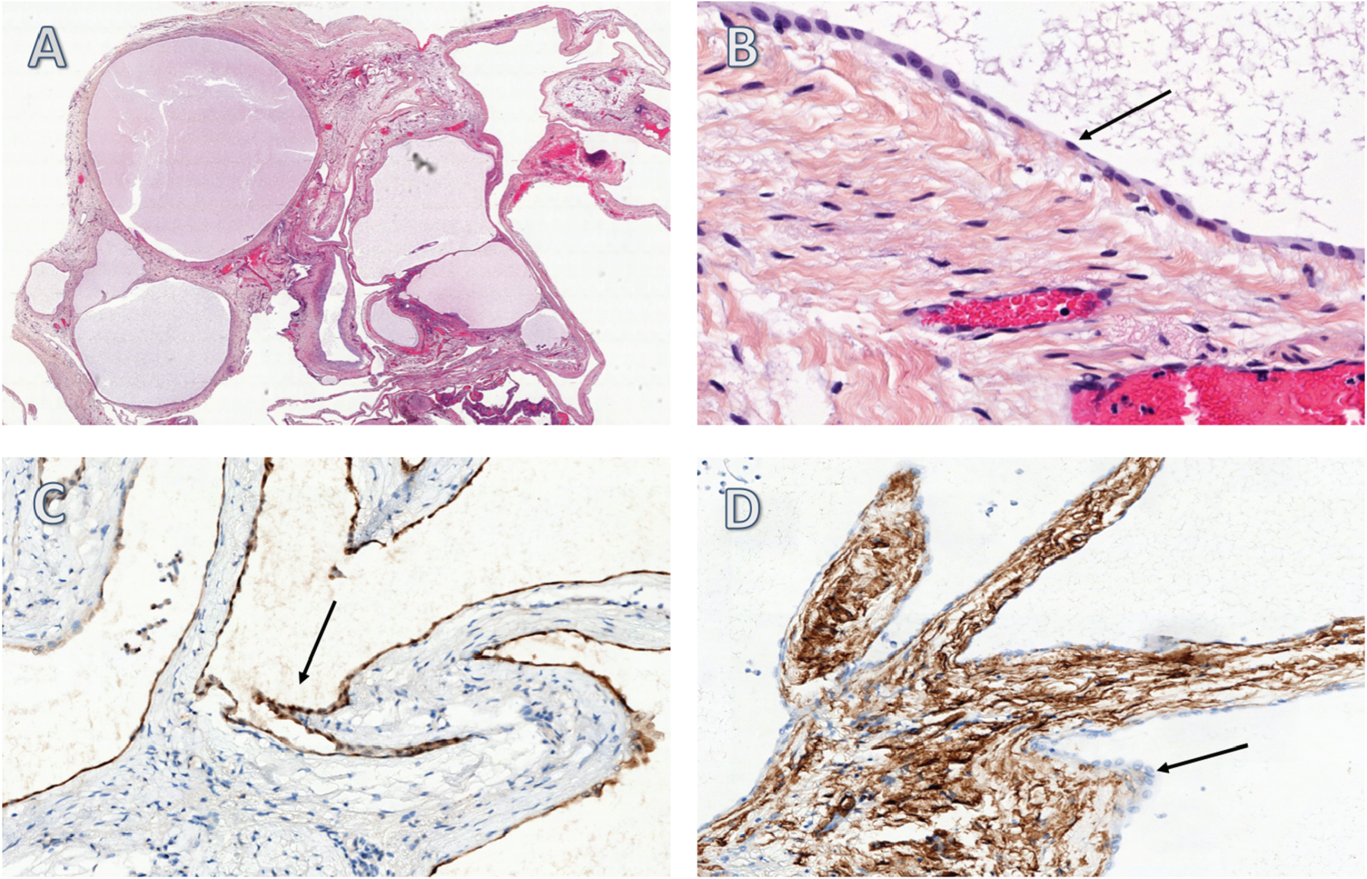
Anatomopathologic aspects of MCPM (x 1) with HES (A). The cystic lesions are lined by a single layer of flattened or cuboidal (arrow) regular mesothelial cells (B). The cells were relatively immunohistochemically positive for calretinin (C) and negative for CD34 (D).

Immunohistochemistry is a necessary step for the definitive diagnosis of MCPM [Figure 3A, B, C, D]. Studies have shown that MCPM biopsies are positive for a series of markers such as the proliferative protein Ki-67 [21], cytokeratin 5/6, calretinin, BAP1, the transcription factor WT-1, and negative for endothelial markers (CD 31, CD 34, and factor VIII) [22] whereas the opposite is true for lymphangioma. Some MCPM were also found positive for the estrogen and progesterone receptors.

## Treatment

Over the last decade, the most common treatment has consisted of a complete resection of the tumor. However, the recurrence rate after resection is approximately 50% after a period of 3 to 27 months (mean 32 months) [23]. No risk factors predicting the recurrence of MCPM have been identified yet.

Alternative treatments and observation such as hormonal therapy, sclerotherapy, and potassium-titanyl-phosphate laser vaporization have been proposed in the recent studies. Hormonal therapy with anti-estrogen drugs like tamoxifen [24] and GnRH agonist [25] can be an alternative to surgery in selected patients with estrogen-dependent neoplasm, as they were showed to be associated with a decrease in cyst volume. Laser vaporization with potassium titanyl-phosphate laser where shown to have a significant efficiency at penetrating the tumor, but its therapeutic efficacy is still unknown [26]. Sclerotherapy consists of injecting povidone–iodine or ethanol through a catheter directly into the cyst. Jeong et al. showed that this therapeutic approach significantly decreases the diameter of the cysts of more than 50%, as well as the abdominal pain, with no signs of recurrence during the follow up period (4 to 60 months) [27].

Due to the high risk of recurrence rate and potential malignant transformation, some studies propose an aggressive approach using CRS with extended peritonectomy associated with HIPEC. The HIPEC protocol usually combines CISPLATIN and DOXORUBICIN. Baratti et al. published the first multi-institutional study with 12 MCPM patients treated by CRS and HIPEC, and showed no recurrence over a follow-up period of 64 months [21]. In addition, a study of 26 MCPM patients with multicystic tumors treated by CRS and HIPEC, showed that they were alive after a median follow-up period of 54 months (range 5 to 129) [28]. Furthermore, Nizri et al. reported long-term outcomes in 19 MCPM patients who underwent 20 CRS and HIPEC procedures. With a median follow-up of 69 months (range 4 to 220), all patients were alive and only 4 patients had recurrence (21%). After 10 years of follow-up, about 80% of the patients remained disease free. This study concludes that CRS and HIPEC for the treatment of MCPM offer low recurrence rate and prolonged survival [29].

The use of the combined laparoscopy CRS – HIPEC may be a promising alternative approach to treat MCPM. Passot et al. included in their study patients with low-grade PMP or MCPM, and compared 8 patients treated by laparoscopy with 8 patients treated by laparotomy. No recurrence has been reported after a median follow-up of 9, 5 months. This cohort study suggests that laparoscopy CRS-HIPEC should be reserved for selected patients with borderline tumors (like MCPM and low-grade PMP) or PCI (Peritoneal Cancer Index) of 10 or less [30]. Moreover, the use of this minimally invasive procedure decreases morbidity and length of hospital stay [31]. The main limitation of laparoscopy is the difficulty to explore the entire abdomen, mesentery, or pelvis.

## Conclusions

In summary, MCPM is a rare benign neoplasm that is associated with no well-defined symptoms, typical clinical, or imaging data. Consequently, the diagnosis remains unclear and the definitive diagnosis requires histology and immunohistochemistry. MCPM has a high risk of recurrence and a significant potential of malignant transformation. Hence, the definition of low-grade or borderline tumors seems more appropriate to describe the lesion. Therefore, a combination of CRS and HIPEC in specialized center is the recommended strategy to decrease the risk of recurrence and increase patient’s overall survival.
